# Proteolytic systems’ expression during myogenesis and transcriptional regulation by amino acids in gilthead sea bream cultured muscle cells

**DOI:** 10.1371/journal.pone.0187339

**Published:** 2017-12-20

**Authors:** Emilio J. Vélez, Sheida Azizi, Dorothy Verheyden, Cristina Salmerón, Esmail Lutfi, Albert Sánchez-Moya, Isabel Navarro, Joaquim Gutiérrez, Encarnación Capilla

**Affiliations:** Departament de Biologia Cellular, Fisiologia i Immunologia, Facultat de Biologia, Universitat de Barcelona, Barcelona, Spain; Universitat Politècnica de València, SPAIN

## Abstract

Proteolytic systems exert an important role in vertebrate muscle controlling protein turnover, recycling of amino acids (AA) or its use for energy production, as well as other functions like myogenesis. In fish, proteolytic systems are crucial for the relatively high muscle somatic index they possess, and because protein is the most important dietary component. Thus in this study, the molecular profile of proteolytic markers (calpains, cathepsins and ubiquitin-proteasome system (UbP) members) were analyzed during gilthead sea bream (*Sparus aurata*) myogenesis *in vitro* and under different AA treatments. The gene expression of calpains (*capn1*, *capn3* and *capns1b*) decreased progressively during myogenesis together with the proteasome member *n3*; whereas *capn2*, *capns1a*, *capns1b* and ubiquitin (*ub*) remained stable. Contrarily, the cathepsin D (*ctsd*) paralogs and E3 ubiquitin ligases *mafbx* and *murf1*, showed a significant peak in gene expression at day 8 of culture that slightly decreased afterwards. Moreover, the protein expression analyzed for selected molecules presented in general the same profile of the mRNA levels, which was confirmed by correlation analysis. These data suggest that calpains seem to be more important during proliferation, while cathepsins and the UbP system appear to be required for myogenic differentiation. Concerning the transcriptional regulation by AA, the recovery of their levels after a short starvation period did not show effects on cathepsins expression, whereas it down-regulated the expression of *capn3*, *capns1b*, *mafbx*, *murf1* and up-regulated *n3*. With regards to AA deficiencies, the major changes occurred at day 2, when leucine limitation suppressed *ctsb* and *ctsl* expression. Besides at the same time, both leucine and lysine deficiencies increased the expression of *mafbx* and *murf1* and decreased that of *n3*. Overall, the opposite nutritional regulation observed, especially for the UbP members, points out an efficient and complementary role of these factors that could be useful in gilthead sea bream diets optimization.

## Introduction

Muscle growth regulation in vertebrates requires an equilibrium among protein synthesis and degradation (proteolysis). This balance acquires special interest in fish, since they mostly present indeterminate growth, and thus increase their muscle mass throughout their life, as well as they are naturally exposed to periods of low food availability, when metabolic mobilization becomes important to survive fasting.

Fish muscle growth differs in many species from other vertebrates in so most mammals can develop muscle mass only by hypertrophy after sexual maturation, whereas fish still continue to increase their muscle mass with important rates of muscle hyperplasia (myogenesis). Understanding on the regulation of fish myogenesis has increased significantly in the last years (reviewed by Fuentes et al. [1]; Johnston et al. [2]; Vélez et al. [3]), as well as the importance of proteolysis on muscle growth *in vivo*; however, the investigation involving the proteolytic systems *in vitro* has been only limited [4–8]. Furthermore, muscle proteolysis research is important in aquaculture because the proteolytic systems play a key role in determining the fish flesh quality. During the *post mortem* period, the muscle tissue is subjected to changes caused by many factors such as temperature, pH or microbial activity, which in conjunction with the action of endogenous proteases can modify muscle properties [5]. Therefore, due to its significance in regulating both, muscle growth and value, to fully elucidate the role of the different proteolytic systems in fish is of utmost importance.

The four chief endogenous proteolytic systems in vertebrates include: cathepsins, calpains, the ubiquitin-proteasome (UbP) and caspases [9, 10]; although the caspases will not be considered in this study as they are mostly linked to cellular apoptosis [11].

The cathepsin family contains several classes of proteases comprising: 1) cysteine proteases (CTSB, L, H, K, S and O), 2) aspartyl proteases (CTSD and E) and, 3) serine proteases (CTSG). Most cathepsins are lysosomal enzymes and part of the autophagic-lysosome system (ALS) involved in cellular degradation. In fact, they are characterized as regulators of an enormous number of biological processes like bone remodeling or angiogenesis, and have been implicated in the development of different pathological conditions (e.g. inflammation and cancer) [12]. In fish, Seiliez and co-workers have recently demonstrated in rainbow trout (*Oncorhynchus mykiss*) myotubes that the ALS is responsible for up to 50% of total protein degradation in contrast to mammals, in which this system appears to be proportionally less important [8].

The calpain system is composed by intracellular proteases that are Ca^2+^-dependent and belong to the papain superfamily of cysteine proteases. The catalytic CAPN1 and CAPN2 subunits bind a common regulatory member, CAPN4 or calpain small subunit (CAPNS) to form an active heterodimer, that has different biological functions during myogenesis depending on the catalytic member [13]. While CAPN1 may be involved in the myogenic regulation via its action on myogenin, ezrin, vimentin and caveolin 3 [14], CAPN2 participates in the fusion of mononuclear myoblasts to multinucleated myotubes in muscle cell cultures [15]. To date, members of this proteolytic system have been characterized in several teleost fish including gilthead sea bream (*Sparus aurata*). In addition in this species, the expression of *capn1* and *capns1a*, was shown to be inversely correlated with muscle texture, indicating they may serve as potential genetic markers of flesh quality [16].

In the UbP system, a large proportion of the proteins intended for degradation in the cell (representing up to 50% in mammals) are tagged by ubiquitination, and then recognized by the 26S proteasome complex, where they are degraded to oligopeptides [9]. Nonetheless in fish, the UbP system is only responsible for 17% of the protein degradation as demonstrated in rainbow trout myotubes [8]. Among the members that conform this system, the muscle specific F-box protein (MAFbx, a.k.a. Atrogin1/Fbox-32) and the muscle RING-finger protein 1 (MuRF1) are key E3 ubiquitin ligases specifically expressed in skeletal, cardiac and smooth muscle that perform multiple functions [17], and have been found up-regulated in situations of muscular atrophy [18]. Similarly, although ubiquitin has multiple functions either proteolytic or non-proteolytic, its expression has been found to increase with age in mammals, which has been related with the poorer healing capacity of the muscle in the elderly [19]. Furthermore, N3 (a.k.a. PSMB4) is a β type proteasome subunit that has been previously used as a proteolysis marker of this degradation system in fish [16, 20–22].

To study skeletal muscle *in vitro* development in mammals, several cell lines have been characterized (e.g. C2C12, L6 or HSkM), but equivalent models are not available in farmed fish, turning primary cultures essential. Therefore, during the last decade primary cultures of myocytes derived from isolated white muscle satellite cells have been established for some economically important fish species, like rainbow trout [23], gilthead sea bream [24], Atlantic salmon (*Salmo salar*) [25], giant danio (*Devario cf*. *aequipinnatus*) [26] and even, zebrafish (*Danio rerio*) [27]. These fish models represent a useful tool to study not only the conserved mechanisms taking place during myogenesis, but also can facilitate the identification of specific-critical factors involved in this process. In this sense, for example the regulation of myogenic development by nutritional factors such as amino acids (AA) has been investigated in several fish species including gilthead sea bream [28, 29]. These studies demonstrated the stimulatory effect of AA on myocytes proliferation and differentiation, as well as the critical negative effect on such processes of lysine limitation. Notwithstanding, information regarding the function of cathepsins, calpains and UbP members on fish myogenesis and how these catabolic systems respond to either AA supplementation or limitation is scarce, and most of the studies reported to date have been performed in salmonids [8, 30, 31].

Thus, the aim of this study was to characterize these 3 main proteolytic systems in gilthead sea bream during *in vitro* myogenesis and the transcriptional modulation of its members by AA to better understand the overall regulation of muscle development and growth in this important farmed species.

## Material and methods

### Experimental animals and ethical statement

The gilthead sea bream were provided by a commercial hatchery in northern Spain (Tinamenor S.L., Pesués, Cantabria). The fish were kept in tanks of 0.4 m^3^ with a closed-water flow circuit at the facilities of the Faculty of Biology at the University of Barcelona. Conditions in the tanks, such as temperature of the sea water (21 ± 1°C), photoperiod (12 h light: 12 h dark) and pH (7.5–8), were kept stable at all times. Twice a day fish were fed *ad libitum* with a commercial diet (Skretting, Burgos, Spain). The animal handling procedures were carried out with the specific approval of the Ethics and Animal Care Committee of the University of Barcelona (permit numbers CEEA 168/14 and DAAM 7749), following the EU, Spanish and Catalan Government-assigned principles and legislations.

### Myocyte cell culture

A total of fifteen independent white muscle satellite cell cultures were performed following the method described previously by Montserrat et al. [24]. Around 40 juvenile fish weighing 5 to 15 g were used for each culture. The fish were sacrificed by a blow to the head, weighed and immediately, their external surfaces were sterilized by immersion in 70% ethanol during 0.5 to 1 min. Then, fish were dissected and the epaxial white muscle tissue was collected in cold Dulbecco’s Modified Eagle’s Medium (DMEM), containing 9 mM NaHCO_3_, 20 mM HEPES, 0.11% NaCl, and 1% (v/v) antibiotic/antimycotic solution, and in this case supplemented with 15% (v/v) horse serum (HS) at a rate of 5 mL/g of tissue. Subsequently, muscle was minced to small fragments and centrifuged (3000 xg, 5 min), washed twice in DMEM and afterwards, the muscle shreds were enzymatically digested with 0.2% collagenase type IA dissolved in DMEM with gentle agitation during 80 min at 21°C. The obtained suspension was centrifuged and the pellet washed with DMEM medium (300 xg, 5 min), resuspended again and triturated by repeated pipetting. After centrifuged once more (300 xg, 5 min), the tissue fragments were digested twice during 20 min at 21°C, with 0.1% trypsin solution prepared in DMEM and gentle agitation. After each digestion the remained fragments were pelleted (300 xg, 1 min) to collect the supernatants, which were pooled and diluted in complete medium (DMEM supplemented with 15% of HS) to block trypsin activity. Then, the supernatant was centrifuged (300 xg, 20 min) and the obtained pellet resuspended, forced to trituration by pipetting and then, the suspension was filtered first on a 100 μm, and subsequently on a 40 μm nylon cell strainer, and finally centrifuged one last time (300 xg, 20 min). Later, the obtained cells were diluted in growth media (DMEM supplemented with 10% fetal bovine serum (FBS) and seeded in six well-plates (9.6 cm^2^/well) at a final density of 1–2 x 10^6^ cells per well. Cultures were kept at 23°C in growth medium with medium change every 2–4 days. To characterize the role of the different proteolytic systems during myogenesis, cell samples for gene and protein expression were taken at days 2, 4, 8 and 12 of culture. These days were chosen because they represent well the different stages of myogenesis, which can be followed according to cell morphology, and are supported by data reported in previous publications [24, 28, 32, 33].

### Experimental treatments

To study the effects of AA recovery, as described previously by Vélez et al. [28], cells at day 4 were first maintained for 12 h with DMEM with 0.02% FBS, and then, starved during 5 h with a medium deficient in AA (medium B: 10% Earle’s Balanced Salt Solution (EBSS, E7510) with 1% MEM vitamins (M6895), 0.9% NaCl and 0.13% bovine serum albumin (BSA)). Next, cells were held 6 h in medium B alone (Control) or supplemented with an AA cocktail (1% MEM Amino Acids Solution (M5550) and 1% MEM Non-essentials Amino Acids Solution (M7145)) before samples were collected. In the case of the leucine or lysine deficiency experiments, as described before by Azizi et al. [29] other 3 different media were prepared (control, without leucine or without lysine) using DMEM/F12HAM (D9785) devoid of leucine and lysine as a base media, and adding 10% FBS, and the missing AA. The concentration of either leucine (24.2 μM) or lysine (24.7 μM) provided by the FBS in each corresponding deficient medium was reduced in a 93.8% respect to the control condition (where total concentration was 389.6 μM and 398.0 μM for leucine and lysine, respectively). In this experiment, the growth medium was replaced with the corresponding media at day 1 of culture for samplings at days 2 and 4, and at day 7 for the sampling at day 8.

All plastic ware were obtained from Nunc (LabClinics, Barcelona, Spain) and all reagents were from Sigma-Aldrich (Tres Cantos, Spain) unless stated otherwise.

### Gene expression

#### RNA extraction and cDNA synthesis

Cell samples for RNA extraction from each independent culture were collected from 3 replicate wells pooled together per sampling point during myogenesis characterization and from 2 replicate wells pooled together per condition in both AA experiments using 1 mL of TRI Reagent Solution (Applied Biosystems, Alcobendas, Spain) and processed following the manufacturer’s instructions. A NanoDrop 2000 (Thermo Scientific, Alcobendas, Spain) was used to determine total RNA concentration and purity. Confirmation of RNA integrity was performed in a 1% (m/v) agarose gel stained with SYBR-Safe DNA Gel Stain (Life Technologies, Alcobendas, Spain). In order to obtain cDNA, 500 ng of the total RNA was first exposed to a DNase I enzyme (Life Technologies, Alcobendas, Spain) to remove all genomic DNA and after reversely transcribed by using a Transcriptor First Strand cDNA synthesis Kit (Roche, Sant Cugat del Valles, Spain) according to the manufacturer’s recommendation.

#### Quantitative real-time PCR (qPCR)

Levels of mRNA transcripts of different cathepsins (*ctsda*, *ctsdb*, *ctsb* and *ctsl*), calpains (*capn1*, *capn2*, *capn3*, *capns1a* and *capns1b*) and UbP members (*mafbx*, *murf*, *n3* and *ub*), as well as the reference genes ribosomal protein S18 (*rps18*), elongation factor 1 alpha (*ef1α*) and beta-actin (*β-actin*) were analyzed according to the MIQE guidelines requirements [34] in a CFX384™ Real-Time System (Bio-Rad, El Prat de Llobregat, Spain). The qPCR reactions were performed using 2.5 μL of iQ SYBR Green Supermix (Bio-Rad, El Prat de Llobregat, Spain), 250 nM of forward and reverse primers (Table 1) and 1 μL cDNA of each sample at the corresponding dilution for an efficient measurement in a final volume of 5 μL. Each run was performed in triplicate using 384-well plates and conditions were the same as those described previously [16]. Briefly, a short initial activation of 3 min at 95°C was followed by 40 cycles of 10 sec at 95°C, 30 sec at 54–61°C (primer dependent, Table 1) and ended with an amplification dissociation analysis from 55 up to 95°C with a 0.5°C increase every 30 sec. Although all the primers have been previously validated [16, 22], a dilution curve with a pooled sample was made before the analyses to confirm reaction specificity, absence of primer-dimers, efficiency of the primers pairs (Table 1) and to determine the appropriate cDNA dilution to work with. Transcript abundance of each studied gene was calculated relative to the geometric mean of the three reference genes (*rps18*, *β-actin* and *ef1α*) since they were all stable (confirmed by the geNorm algorithm) using the method described by Pfaffl [35] with the Bio-Rad CFX Manager 3.1 software.

**Table 1:** Primer sequences used for qPCR.

Gene	Primer sequences (5’-3’)	Accession No.	Ta (°C)	Amplicon (bp)	E (%)
*ef1a*	**F:**CTTCAACGCTCAGGTCATCAT **R:**GCACAGCGAAACGACCAAGGGGA	AF184170	60	263	96.6
*β-actin*	**F:**TCCTGCGGAATCCATGAGA **R:**GACGTCGCACTTCATGATGCT	X89920	68	50	98.3
*rps18*	**F:**GGGTGTTGGCAGACGTTAC **R:**CTTCTGCCTGTTGAGGAACCA	AM490061.1	60	160	97.7
*ctsda*	**F:**CCTCCATTCACTGCTCCTTC **R:**ACCGGATGGAAAACTCTGTG	AF036319	56	107	102.1
*ctsdb*	**F:**AAATTCCGTTCCATCAGACG **R:**CTTCAGGGTTTCTGGAGTGG	KJ524456	56	131	95.6
*ctsb*	**F:**GCAGCCTTCCTGTTATTGG **R:**AGGTCCCTTCAGCATCGTA	KJ524457	57	185	95.0
*ctsl*	**F:**ACTCCTTGGGCAAACACA **R:**CCTTGAACTTCCTCTCCGT	DQ875329	54	116	94.5
*capn1*	**F:**CCTACGAGATGAGGATGGCT **R:**AGTTGTCAAAGTCGGCGGT	KF444899	56	114	103.2
*capn2*	**F:**ACCCACGCTCAGACGGCAAA **R:**CGTTCCCGCTGTCATCCATCA	KF444900	61	405	91.3
*capn3*	**F:**AGAGGGTTTCAGCCTTGAGA **R:**CGCTTTGATCTTTCTCCACA	ERP000874	56	113	97.2
*capns1a*	**F:**CGCAGATACAGCGATGAAAA **R:**GTTTTGAAGGAACGGCACAT	KF444901	56	92	100.2
*capns1b*	**F:**ATGGACAGCGACAGCACA **R:**AGAGGTATTTGAACTCGTGGAAG	ERP000874	56	51	99.7
*mafbx*	**F:**GGTCACCTGGAGTGGAAGAA **R:**GGTGCAACTTTCTGGGTTGT	ERA047531	60	158	94.3
*murf1*	**F:**GTGACGGCGAGGATGTGC **R:**CTTCGGCTCCTTGGTGTCTT	FM145056	60	50	98.5
*n3*	**F:**AGACACACACTGAACCCGA **R:**TTCCTGAAGCGAACCAGA	KJ524458	54	118	99.1
*ub*	**F:**ACTGGCAAGACCATTACCTT **R:**TGGATGTTGTAGTCGGAAAG	KJ524459	54	160	97.2

F: forward; R: reverse; Accession No.: GenBank accession numbers; Ta: annealing temperature; Amplicon: product size (base pairs); E: qPCR efficiency.

### Protein expression

#### Protein extraction

Protein isolation was carried out from the same samples taken at different time points during myogenesis and obtained from triplicate wells pooled together from each independent culture using the interphase and organic phase produced during the RNA isolation and following the TRI Reagent Solution manufacturer protocol. The protein pellets were resuspended with 60 μL of RIPA buffer (Tris-HCl 50 mM pH 7.4, NaCl 150 mM, EDTA 2 mM, NP-40 1%, SDS 0.1% and Na-deoxycholate 0.5% plus protease inhibitor cocktail P8340) and homogenated with a Pellet pestle (Sigma-Aldrich). Then, the samples were kept for 1 h in an orbital at 4°C and centrifuged at 15,000 xg for 30 min at 4°C. Finally, the supernatant was transferred to a new tube and stored at -80°C until further analysis.

#### Western blot analysis

Protein concentration of each sample was measured using the Bradford assay, with BSA as reference protein. Four to ten μg of soluble fraction protein were subjected to a SDS-PAGE gel electrophoresis on a 12% acrylamide gel (1 h 30 min at 125 V) following the procedure previously described [28]. After blocking with 5% non-fat milk in washing buffer (20 mM Tris HCl, 150 mM NaCl, 0.05% Tween 20, pH 7.6), the membranes were incubated with the primary antibodies diluted in washing buffer overnight at 4°C. The primary polyclonal antibodies used were CAPN1 (sc-7530), CTSD (sc-6486), CTSL (sc-6501) and MAFbx (sc-33782), all from Santa Cruz Biotechnology (Santa Cruz, CA, US). CAPN1, CTSD and CTSL were used at 1:200 and MAFbx at 1:400 final concentration. Afterwards, the membranes were washed and incubated with the respective secondary antibodies (sc-2020 and sc-2004, also provided by Santa Cruz Biotechnology) in a 5% non-fat milk washing buffer solution at a final concentration of 1:10000. After washing, an enhanced chemiluminescence kit (Pierce ECL WB Substrate, Thermo Scientific, Alcobendas, Spain) was used to develop the bands. When required, the membranes were stripped for 15 min at 65°C and 30 min at 37°C on a roller with a commercial stripping buffer (Restore Western Blot Stripping Buffer, Thermo Scientific). The software ImageJ (National Institutes of Health, Bethesda, MD, USA) was used to quantify the obtained bands by densitometry. Since the band corresponding to the immature form of cathepsin D (CTSD imm) was stable during myogenesis, the expression of this protein was used as a loading control to normalize the expression of all the other proteins analyzed.

### Statistical analyses

IBM SPSS Statistics v.20 was used to analyze the data. The results are presented as means ± SEM. All the raw data underlying the obtained results can be found in the S1 File. A Shapiro-Wilk test was performed to analyze the normality of the data and homogeneity of the variances was tested with a Levene’s test. When normality existed, data was subjected to a one-way ANOVA followed by a Tukey or Dunnet T3 *post-hoc* test depending if respectively there was homogeneity of variances or not. Nevertheless, when normality was not assumed, the non-parametric Kruskal-Wallis test was used followed by a Mann-Whitney U test. Similarly, correlations between mRNA and protein levels were established either with a Spearman’s rank correlation coefficient (ρ) or a Pearson correlation (PC). Differences were considered significant at p<0.05.

## Results

### Gene and protein expression profiles of proteolytic markers during myogenesis

**Fig 1:**
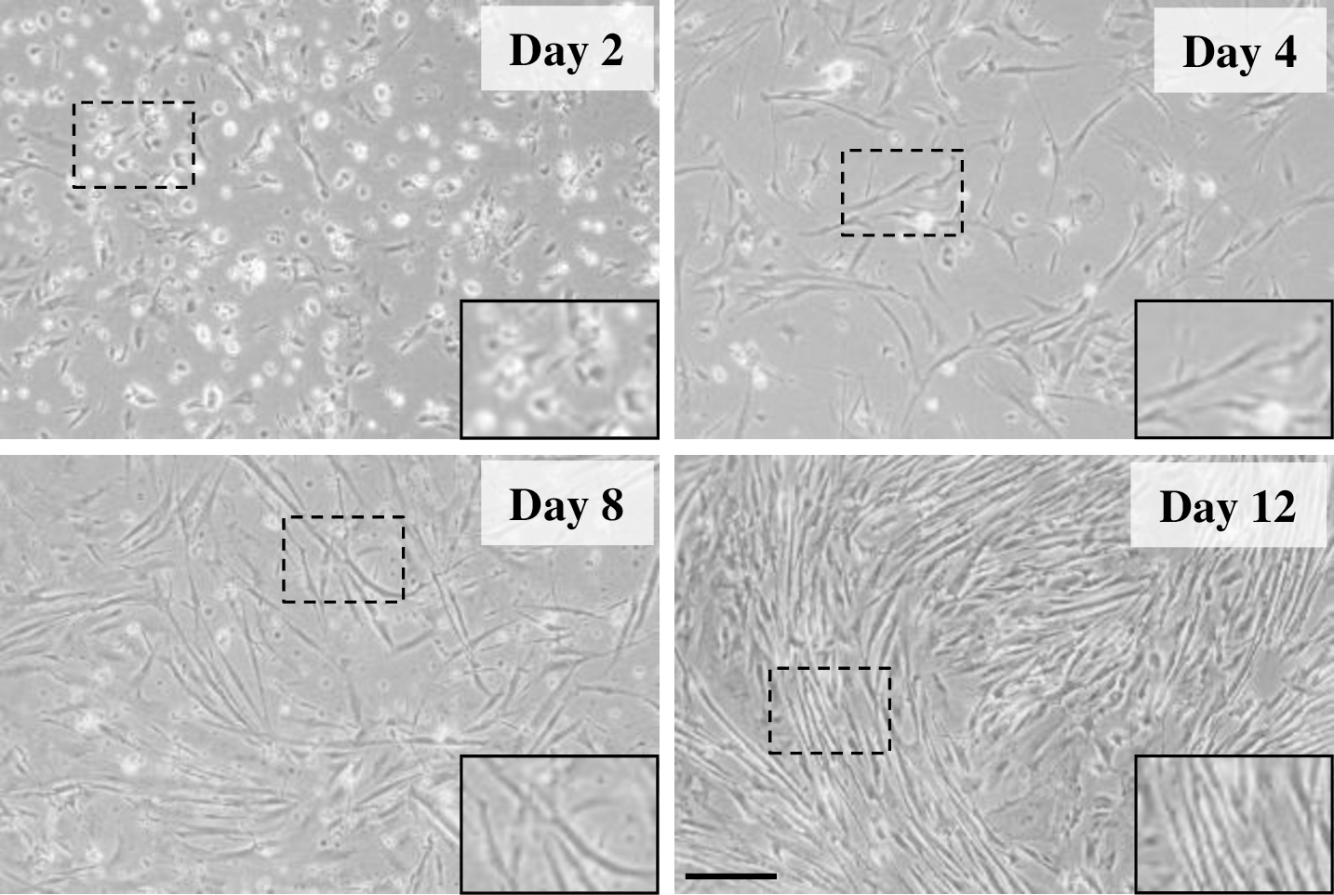
Representative images of gilthead sea bream cultured myocytes at days 2, 4, 8 and 12 of development. Images were taken with an EOS 1000D Canon digital camera coupled to an Axiovert 40C inverted microscope (Carl Zeiss, Germany). Objective: 10x. Scale bar: 50 μm. Insets in each image are enlarged views of cells from each panel.

The transcriptional profile of several members of the three endogenous proteolytic systems was studied in gilthead sea bream myocytes at days 2, 4, 8 and 12 of culture. On day 2, activated mononucleated myoblasts cells are undergoing active proliferation to become myocytes (day 4). Then, cells subsequently differentiate and fuse to form small myotubes (day 8), and later on (day 12) some large myotubes can be observed (Fig 1).

**Fig 2:**
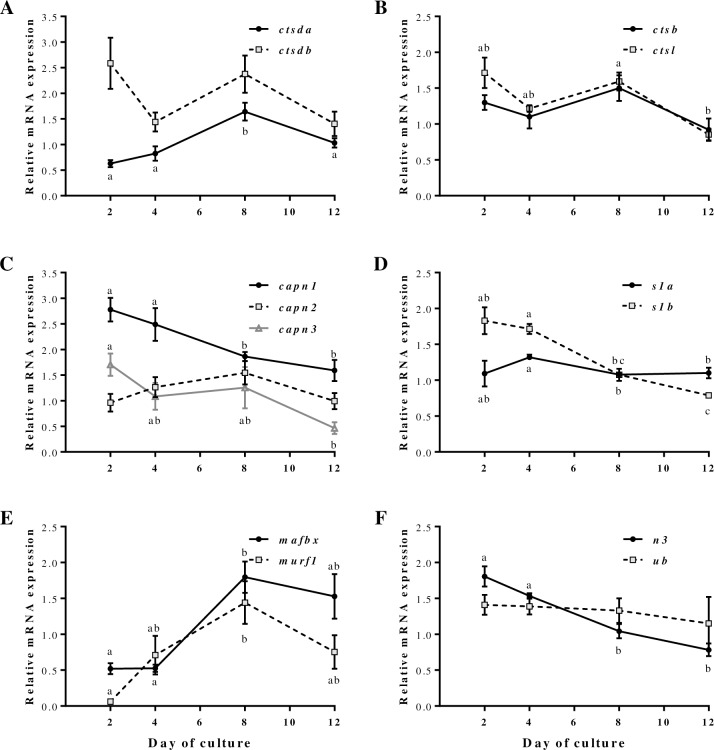
Cathepsins, calpains and ubiquitin-proteasome members mRNA levels during *in vitro* myogenesis in gilthead sea bream. Quantitative gene expression relative to the geometric mean of *β-actin*, *rps18* and *ef1α* of (**A**) *ctsda* and *ctsdb*, (**B**) *ctsb* and *ctsl*, (**C**) *capn1*, *capn2* and *capn3*, (**D**) *capns1a* (*s1a*) and *capns1b* (*s1b*), (**E**) *mafbx* and *murf1*, and (**F**) *n3* and *ub*. Results are shown as mean ± SEM (n = 4 independent cultures). Different letters indicate significant differences at p<0.05.

Concerning the cathepsins gene expression, although the profile of the two *ctsd* paralogs throughout the culture was quite similar, *ctsda* showed increased mRNA expression at day 8 compared to the other days, while the changes observed in the expression of *ctsdb* were not significantly different (Fig 2A). *ctsb* and *ctsl* showed as well a similar profile to that of *ctsdb*, with *ctsl* being significantly down-regulated in the last stage of myocyte differentiation (Fig 2B). Regarding calpains gene expression, a significant decrease during myogenesis was observed for *capn1*, *capn3* and *capns1b*, while *capn2* and *capns1a* remained stable (Fig 2C and 2D). In the case of the UbP members, the gene expression data showed that while ubiquitin E3 ligases (*mafbx* and *murf*) significantly increased up to day 8 to decrease afterwards (Fig 2E), *ub* remained stable and the proteasome beta-type subunit *n3* was significantly decreased along with myogenesis (Fig 2F).

**Fig 3:**
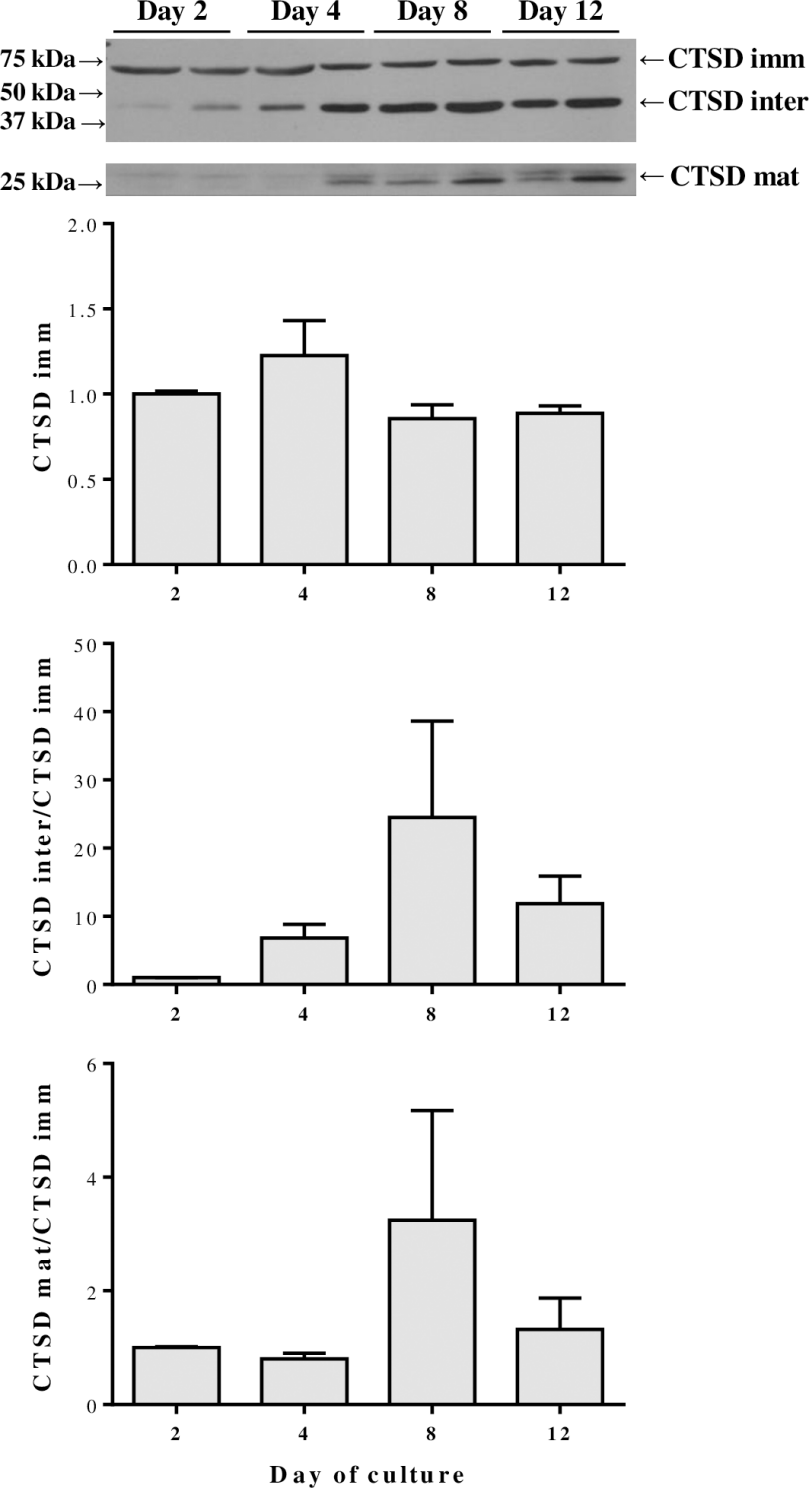
Cathepsin D protein abundance in gilthead sea bream during *in vitro* myogenesis. Representative Western blot showing the immature (top, CTSD imm), intermediate (middle, CTSD inter) and mature (bottom, CTSD mat) forms at days 2, 4, 8 and 12 of myocytes culture. The densitometric data for CTSD inter and CTSD mat was normalized to the corresponding CTSD imm band. Results are shown as mean ± SEM (n = 3 independent cultures). Note: Although all three bands are from the same Western blot, the mature form is shown separated because the image comes from a longer exposed film for better visualization.

**Fig 4:**
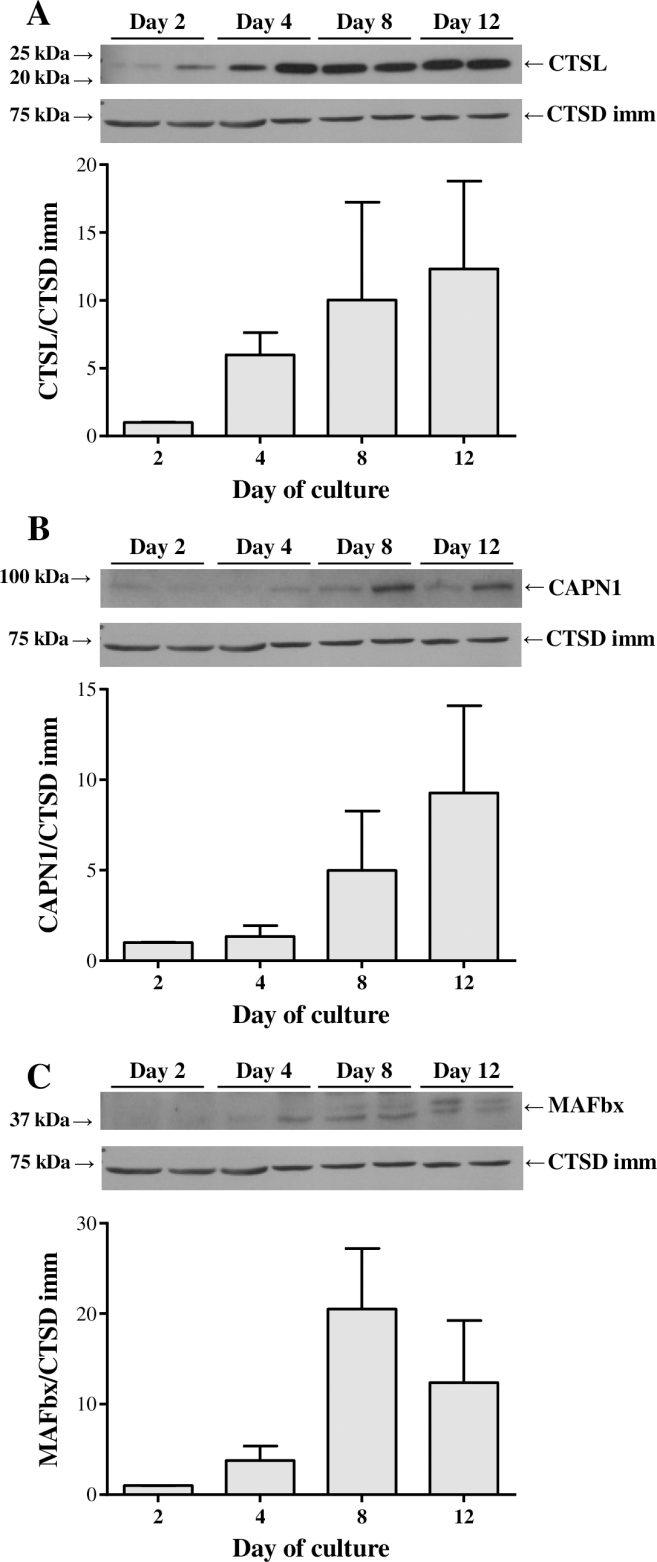
Cathepsin L, calpain 1 and MAFbx protein abundance in gilthead sea bream cultured myocytes. Representative Western blots from (**A**) CTSL, (**B**) CAPN1 and (**C**) MAFbx at days 2, 4, 8 and 12 of myocytes culture development. The densitometric data was normalized relative to the corresponding cathepsin D immature form (CTSD imm). Results are shown as mean ± SEM (n = 3 independent cultures).

In contrast to the gene expression results, the immunoblotting data did not show any significant differences. In this sense, the protein levels of the immature form of CTSD remained very stable throughout myocyte differentiation, although both the mature and intermediate enzymes showed a tendency to increase at day 8 (Fig 3). MAFbx presented as well a peak on protein expression at day 8 of culture (Fig 4C), whereas both CTSL and CAPN1 were gradually increased reaching a maximum of expression at day 12 (Fig 4A and 4B).

### Correlation between gene and protein expression of proteolytic markers during myogenesis

**Fig 5:**
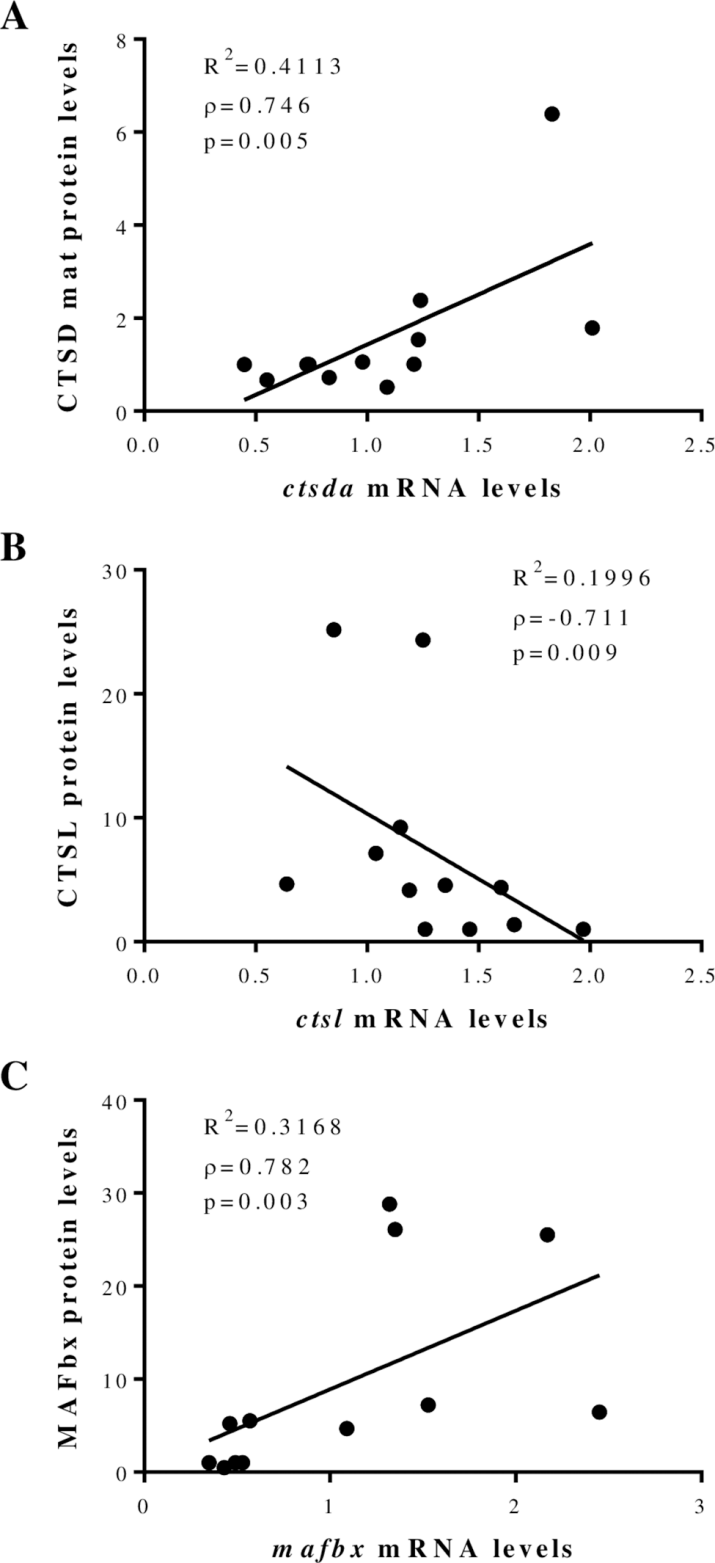
Correlations between mRNA and protein levels of some cathepsins and ubiquitin-proteasome members in gilthead sea bream during *in vitro* myogenesis. Significant correlations between mRNA relative expression and protein abundance in cultured myocytes between (**A**) cathepsin Da (*ctsda*) with cathepsin D mature form (CTSD mat), (**B**) cathepsin L (*ctsl*) with CTSL and (**C**) muscle atrophy F-box (*mafbx*) with MAFbx. Data are from n = 3 independent cultures. The R^2^ of the linear regression, the Spearman’s rank correlation coefficient (ρ) and the p-value are shown.

Despite the absence of significant changes on protein expression during myogenesis, when the data were plotted against the corresponding gene expression levels, a significant positive correlation was found between *ctsda* and mature CTSD (Fig 5A), *ctsda* and intermediate CTSD (R^2^ = 0.4399, PC = 0.662, p = 0.019; S1A Fig) and *mafbx* with MAFbx (Fig 5C). On the other hand, a significant negative correlation was found between *ctsl* mRNA and CTSL protein levels (Fig 5B), whereas also a negative but non-statistically different correlation was found between *capn1* and CAPN1 (R^2^ = 0.1116, ρ = -0.500, p = 0.098; S1B Fig).

### Proteolytic genes expression regulation by recovery or deficiency in selected amino acids

**Fig 6:**
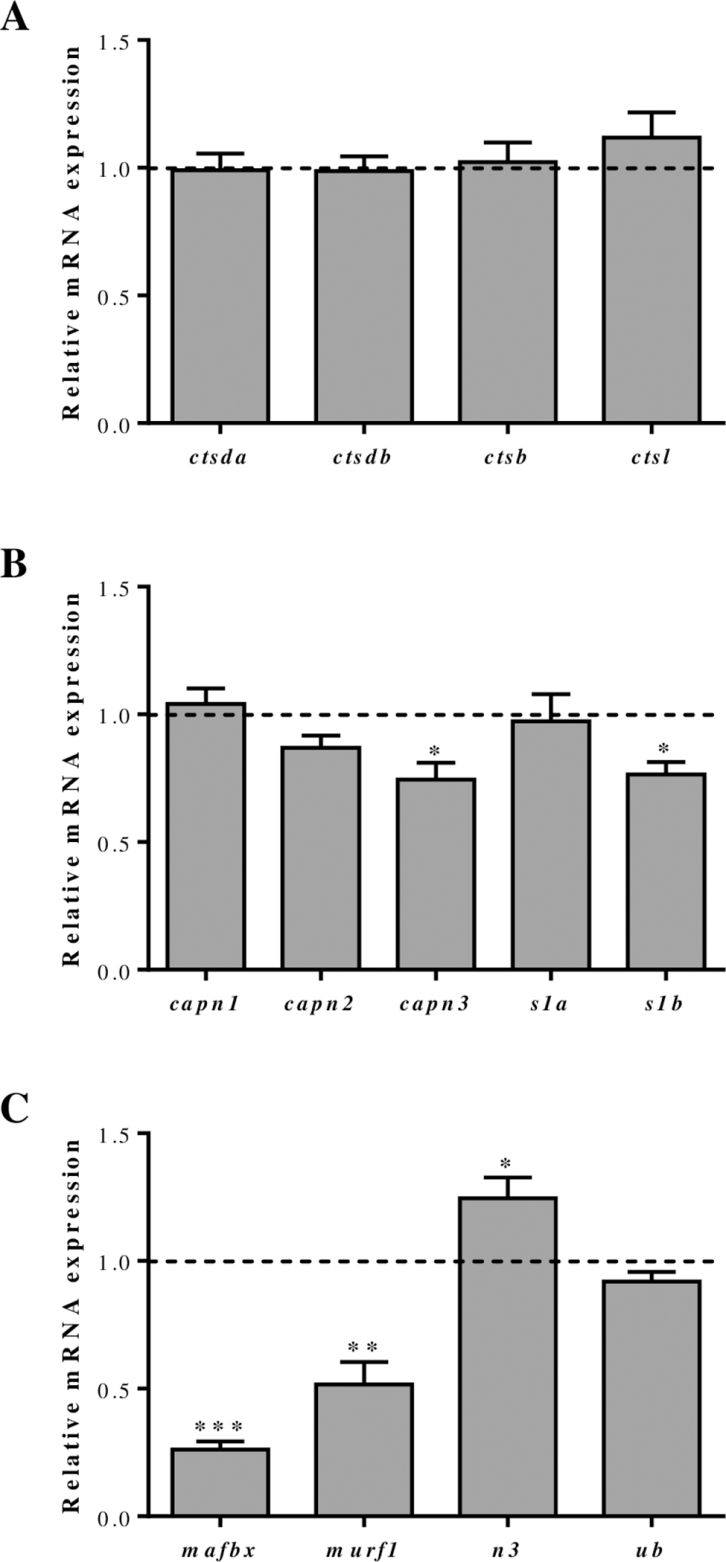
Effects of amino acids (AA) recovery on proteolytic molecules gene expression in gilthead sea bream cultured myocytes. Quantitative gene expression of (**A**) cathepsins (*ctsda*, *ctsdb*, *ctsb*, *ctsl*), (**B**) calpains (*capn1*, *capn2*, *capn3*, *capns1a* (*s1a*), *capns1b* (*s1b*)) and, (**C**) UbP members (*mafbx*, *murf1*, *n3*, *ub*) relative to the geometric mean of *ef1α* and *rps18* in day 4 cultured myocytes supplemented with a cocktail of AA for 6 h after a 12 h starvation period. Results are shown as fold change relative to the control condition (cells maintained without AA for the 18 h period including starvation and treatment), represented by the dotted line. Mean ± SEM (n = 4–7 independent cultures). Asterisks indicate significant differences compared to the control (*: p<0.05; **: p<0.01; ***: p<0.001).

The expression of all cathepsin genes studied remained unchanged when the culture medium was supplemented with a cocktail to recover the AA levels at day 4 (Fig 6A). Similarly, differences were not observed for the calpains *capn1*, *capn2* and *capns1a* (Fig 6B). Contrarily, AA recovery caused a significant decrease on *capn3* and *capns1b* gene expression (Fig 6B) and the same effect was found for *mafbx* and *murf1* (Fig 6C). Nevertheless, this response to recovered AA was not general to all the UbP genes because *ub* was not affected and *n3* was significantly increased.

**Fig 7:**
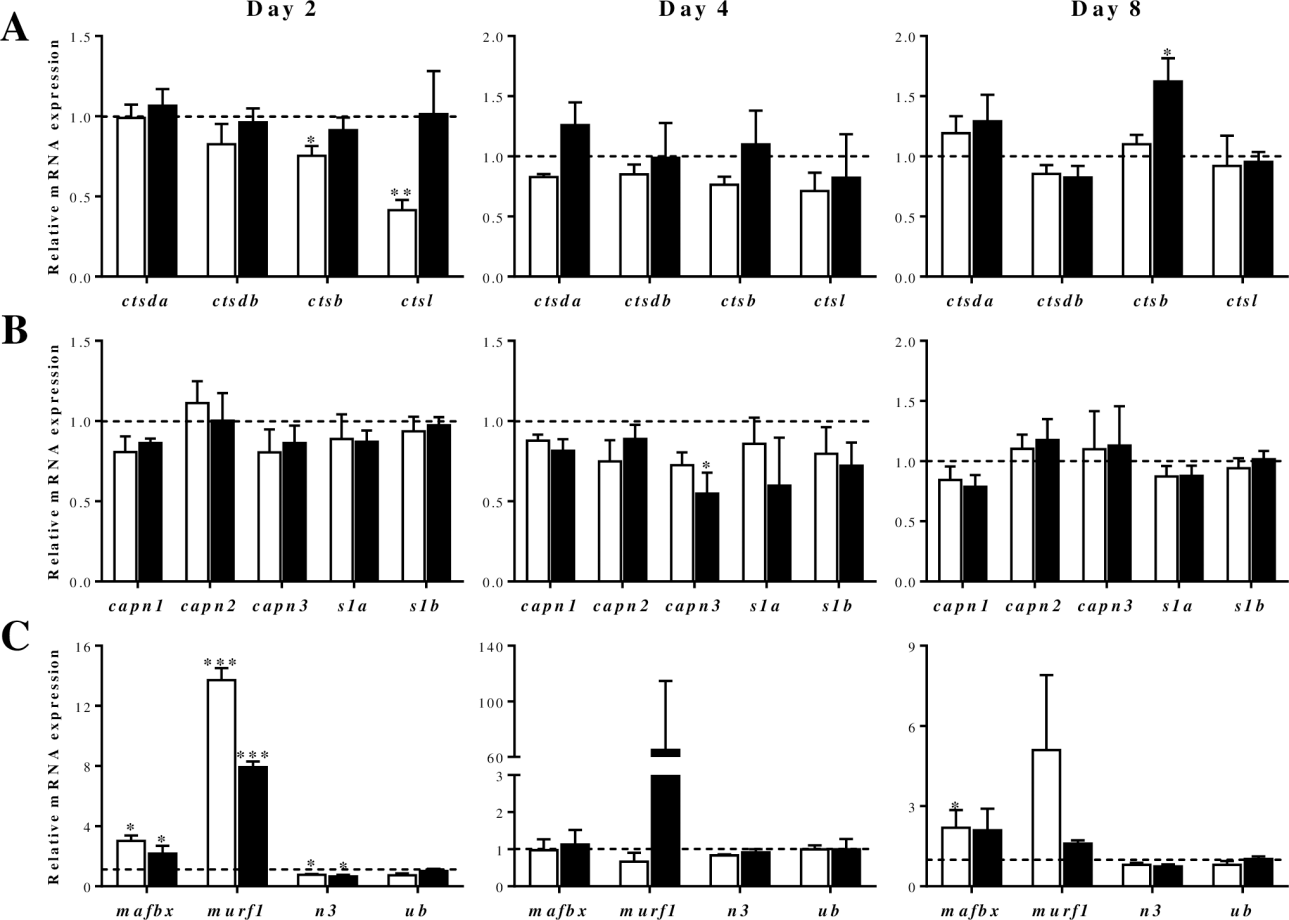
Effects of leucine or lysine deficient media on proteolytic molecules gene expression in gilthead sea bream cultured myocytes. Quantitative gene expression of (**A**) cathepsins (*ctsda*, *ctsdb*, *ctsb*, *ctsl*), (**B**) calpains (*capn1*, *capn2*, *capn3*, *capns1a* (*s1a*), *capns1b* (*s1b*)) and (**C**), UbP members (*mafbx*, *murf1*, *n3*, *ub*) relative to the geometric mean of *ef1α* and *rps18* in myocytes at days 2, 4 or 8 of culture after incubation from day 1 (for samples at days 2 and 4) or day 7 (for samples at day 8) with a growth medium deficient in leucine (open bars) or lysine (filled bars). Data are shown as fold change relative to control condition (growth medium without AA deficiencies), represented by the dotted line. Mean ± SEM (n = 3–4 independent cultures). Asterisks indicate significant differences compared to the control at each time (*: p<0.05; **: p<0.01; ***: p<0.001).

Next, the deficiency of leucine or lysine on the proteolytic gene markers expression through *in vitro* myocytes development was examined (Fig 7). Deficiency in leucine significantly decreased *ctsb* and *ctsl* gene expression in day 2 myocytes (Fig 7A). Otherwise, lysine deficiency did not provoke such an inhibitory effect and contrarily at day 8 *ctsb* expression resulted significantly increased. Furthermore, AA limitation provoked little effects in the gene expression of calpains and only *capn3* was significantly decreased at day 4 in lysine deficient medium (Fig 7B). Among the UbP genes, *mafbx* and *murf1* were the most affected (Fig 7C) with significant up-regulation at day 2 in response to both deficiencies, and at day 8 for *mafbx* when incubated in a medium deficient in leucine. Moreover, *ub* gene expression was not affected at any time upon any condition, while *n3* was significantly decreased after two days in both AA deficiencies.

## Discussion

The first objective of this study was to analyze the mRNA and protein levels of various cathepsins, calpains and UbP members throughout *in vitro* myogenesis in gilthead sea bream in order to unravel the phase in which those systems are required for the adequate progression of the process. Second, we assessed the involvement of AA regulating the gene expression of these three catabolic systems’ members to define how crucial they are and to consider that for fish feeds formulation.

### Characterization of proteolytic markers gene and protein expression during gilthead sea bream myogenesis

The comparison of protein and gene expression of selected proteolytic members revealed the intricate control of these factors. In this sense, correlation analysis for cathepsin D and MAFbx confirmed a parallelism between gene and protein levels, while in the case of cathepsin L and calpain 1 the results indicated an opposite pattern. Such negative correlation could indicate a complex network of mRNA regulation at different levels: transcription, translation and degradation. The same opposite pattern for calpain 1 was observed during L8 rat myoblast fusion [36, 37] with increase of CAPN1 protein abundance in the maturation phase as in this study. Besides, it is interesting to note that even without showing significant differences, most of the molecules studied presented their highest protein levels at the end of myogenesis, suggesting an active role for these enzymes in muscle stabilization/consolidation. This means that these proteolytic molecules might be essential for the remodeling that occurs during muscle formation (i.e. breaking of the cytoskeletal/plasma membranes linkages necessary to create points for myoblast fusion [13, 38]).

Concerning gene expression, the data suggest that cathepsins would have greater importance during the early differentiation phase of myogenesis. In agreement with that, Colella et al. [37] found that *ctsb* gene expression decreased after fusion of myotubes in the L6 rat myogenic line, and Ebisui et al. [39] that the differentiation of C2C12 myoblasts involved up-regulation of lysosomal cathepsins. Contrarily, in chick myoblasts, *ctsb* showed the highest mRNA levels at the proliferative phase [40]. In fish, little information exists, but during salmon myocytes development, Bower and Johnston [41] described the increase of *ctsl1* expression with a peak at day 8, followed by a decrease at day 11 as in our study, and a new increase later at days 17 or 20, suggesting overall a relevant role for CTSL1 in differentiation and formation of myotubes.

Regarding calpains, in the present study expression of *capn1*, *capn3* and *capns1b* decreased progressively during myogenesis but *capn2* and *capns1a* remained stable. Similarly, Nakashima et al. [40] found in chick myoblasts a progressive decrease of *capn1* gene expression through *in vitro* development; whereas in rat muscle primary culture, Stockholm et al. [42] found that *capn1* and *capn2* increased while *capn3* decreased, indicating that the various calpains can be regulated in an opposite way as it occurs in our model. Moreover, Van Ba and Inho [43] also demonstrated that CAPN1 is involved in proliferation and survival during myogenesis in bovine muscle cells. Overall these data indicate that although differences exist among the different vertebrate groups, the main trend is to observe higher gene expression of calpains at the early myogenic stages.

The E3 ubiquitin ligases, MAFbx and MuRF1, are important members of the UbP system. There is evidence that MuRF1 is necessary for the initiation and stabilization of myogenesis [44], being its actions located mainly in the cytoplasm of muscle cells, where it recognizes myofibrillar proteins, such as myosin heavy chain (MHC), and targets them for breakdown [10, 45]. In gilthead sea bream, *mhc* gene expression increased up to day 9 in cultured myocytes and became stable afterwards [32], which is parallel to *murf1* expression and supports also in this species the functional relationship of these molecules. On the other hand, MAFbx is essential for myogenic stem cell function in adult skeletal muscle, as it identifies and targets for ubiquitination several transcription factors with key roles in the control of skeletal muscle development (i.e. myogenic differentiation 1 (MyoD1) or myogenin) [45–47]. García de la serrana et al. [32] found for *myod2* expression in gilthead sea bream myocytes a profile opposite to *mafbx*. This opposed relationship can be explained by the fact that at the start of development the stem cells have to determine their fate and so *myod* levels have to be high, while contrarily, when muscle cells become differentiated, MyoD is no longer needed, and its expression decreases probably due to the up-regulation of *mafbx*. Overall, the profiles of both E3 ligases are similar, which agrees with the findings of Spencer et al. [44] and Perera et al. [48] in mouse skeletal muscle and C2C12 cells, respectively demonstrating that *murf1* is required for myoblast differentiation and myotube fusion, pointing out very well the conserved role of this UbP molecule as well in muscle development.

Concerning the other members of the UbP system, it is well accepted that *n3* is a good marker of proliferation [49, 50], which is in agreement with it showing the same pattern of gene expression as that reported for the proliferation marker *pcna* in gilthead sea bream [29, 32]. Finally, Nakashima et al. [40] observed a significant reduction on *ub* gene expression during chicken myoblast differentiation, although we found it unaltered in gilthead sea bream myocytes. In support of this absence of changes in gene expression, Kimura and Tanaka [51] suggested that ubiquitin plays multiple roles controlled by complex regulatory mechanisms to actually maintain its levels stable.

In summary, as far as we know, the present study shows for the first time in cultured fish myocytes the expression of several proteolytic members that seems to be in agreement with a more relevant role of calpains during the proliferative phase of myogenesis and of cathepsins and the UbP system in muscle cells differentiation. This in concordance with the more anabolic aspect of calpains in comparison to cathepsins and the UbP system, since they do not degrade proteins up to small peptides or AA, but only disassemble the sarcomeric structure of the muscle [10, 13, 15, 44]. Moreover, the expression of *ctsb*, *ctsl*, *ctsdb*, *mafbx* and *n3* was reported greater in the muscle of fingerlings than in juvenile or adult gilthead sea bream, pointing out a major role for these two endogenous systems (cathepsins and UbP) when the myogenic process is more active [22].

### Regulatory effects of recovery or deficiency of selected AA in proteolytic markers gene expression in gilthead sea bream myocytes

Previous studies have demonstrated in gilthead sea bream that almost all cathepsins and UbP system-related genes are up- and down-regulated during fasting and refeeding, respectively [22], and similar results were observed, although to a lesser extent, with regards to calpains [16]. Besides, it has been shown that forced swimming provokes in gilthead sea bream up-regulation of cathepsins and UbP members [38], supporting that muscle remodeling is taking place under both catabolic and anabolic conditions. In this sense, we have found now in myocytes of the same species that specifically the AA seem to have an important role controlling proteolytic systems, although mostly the expression of UbP members.

In agreement with that, Cleveland and Weber [4] found that *ctsd* and *ctsl* expression was not affected by a leucine treatment in rainbow trout myocytes; while contrarily, lysine supplementation had an inhibitory effect on ALS activity in C2C12 myotubes [52]. With regards to calpains, response to AA in this study was observed only for *capn3* and *capns1b*, the same genes modified in response to fasting and refeeding in the same species [16]. In the case of halibut (*Hippoglossus hippoglossus*) and channel catfish (*Ictalurus punctatus*), skeletal muscle *capn3* mRNA was at its lowest level during fasting, and highest in refeeding [53, 54], providing overall these data an evidence for species-specific differences concerning the activity of this gene. Notwithstanding, considering that calpain 3 is a muscle specific regulator of other calpains’ expression and activity, as well as its levels have been correlated with bovine and ovine muscle tenderness [55, 56], these variable responses in fish deserve to be further investigated.

Moving to the expression of UbP genes affected by AA, it is interesting to emphasize that in our study, AA levels recovery decreased the expression of *mafbx* and *murf1* but increased *n3* whereas contrarily, leucine and lysine deficiencies stimulated, mainly at day 2, *mafbx* and *murf1* expression while inhibiting *n3*. These results suggest that both MAFbx and MuRF1 could be increasing the amount of proteins sent to the proteasome when AA are lacking; however, the opposite response of *n3* and the stable *ub* expression might be indicating that the flux of ubiquitinated proteins through the proteasome is constrained (or slowed down). Then, these proteins would be probably degraded by autophagy, as it has been observed in mammals, demonstrating that there is an important cross-talk regulation within the proteolytic systems [56]. This hypothesis makes even more sense in fish, in which in contrast to mammals, the ALS is responsible for around two to three times more protein degradation than the UbP system [8].

Furthermore in *in vitro* models, AA limitation increases proteolysis in an UbP-dependent manner in C2C12 myotubes, although an increase in AA or leucine alone down-regulates protein degradation and the expression of components of the UbP pathway [57]. However, also in C2C12 cells, the expression of *murf1* was not affected after incubation with lysine [52]. Similarly, in rainbow trout myocytes, leucine supplementation did not affect *murf1* while serum deprivation increased the expression of the ubiquitin ligases *mafbx*, *fbx25* and *murf1* [4]. In the case of salmon muscle cells, an starving of AA caused down-regulation of *mafbx* [31], whereas in a previous study analyzing two different splice variants in the same cell model, it was demonstrated that serum and AA starvation resulted in a 6-fold increase in the expression of *mafbx-α*. This isoform expression declined subsequently in response to an AA treatment [58], but *mafbx-β* appeared to be less sensitive to AA since its expression remained similar to the control, and only was altered when insulin or insulin-like growth factors (IGFs) were present in the culture media. Probably, this differential response between isoforms is due to their specific roles during salmon *in vitro* myogenesis, where *mafbx-α* gene expression is highest in differentiated myotubes (similarly to our data), and *mafbx-β* mRNA is more abundant at myoblast stage [58]. Moreover, serum depletion and specifically AA withdrawal in rainbow trout myocytes induced the expression of the autophagy-proteasome genes (*lc3b*, *gabarapl1*, and *atg4b*) [7, 30], suggesting an important role for the AA released by muscle mobilization during fasting, to regulate proteolytic genes.

In this sense, considering our experimental model, Vélez et al. [28] after AA recovery found increases on proliferation, differentiation and protein synthesis markers such as *pcna*, *myogenin*, *tor*, *4ebp1* and *70s6k*, while the expression of *foxo*, a factor involved in the activation of the proteolytic pathway, remained unaffected. After 2 days of leucine limitation, Azizi et al. [29] found that the expression of the AA deficiency indicator *chop* was increased, whereas *4ebp1* and *foxo* diminished. Furthermore, after 8 days of lysine deficiency, an increased expression of other two AA-limitation markers (i.e. *atf4* and *as*) was observed; and also, a decrease in important proteogenic/anabolic pathways’ molecules including members of the IGF system (i.e. *pcna*, *igf-1*, *igf-2*, *igf-1rb*, *akt*, *erk* and *70s6k*) [29]. These data confirm an overall negative effect of the reduced AA levels, especially lysine, on protein turnover and thus, muscle growth in gilthead sea bream, which is supported by our results.

The present study provides new information about the potential role of key members of the endogenous proteolytic systems (cathepsins, calpains and UbP) in gilthead sea bream cultured muscle cells. We can suggest that there is a functional distribution between the different proteolytic system molecules throughout the *in vitro* development of muscle cells at least until the phases of myocyte differentiation and small myotube formation (day 8). Besides, it is interesting to note the up-regulation of *mafbx* and *murf1* in response to AA deficiencies and their down-regulation with AA recovery and the reverse response of *n3*, pointing out to an efficient and complementary role of these UbP system members to AA supply.

In summary, the research on the function of proteolytic systems in fish offers interesting information on the evolution of myogenesis regulation and the effects of AA on such process that can have valuable application in aquaculture in order to optimize diet composition for this species.

## Supporting information

S1 File Raw data. (XLSX)

S1 Fig Correlations between mRNA and protein levels of some cathepsins and calpains in gilthead sea bream during *in vitro* myogenesis. (A) cathepsin Da (*ctsda*) with cathepsin D intermediate form (CTSD inter), and (B) calpain 1 (*capn1*) with CAPN1. Data are from n = 3 independent cultures. The R^2^ of the linear regression, the Spearman’s rank correlation coefficient (ρ) and the p-value are shown.
(TIF)
