# Supplementary figures and images for: Proteolytic systems’ expression during myogenesis and transcriptional regulation by amino acids in gilthead sea bream cultured muscle cells
Source: PLoS One. 2017 Dec 20;12(12):e0187339. doi: 10.1371/journal.pone.0187339 (PMC5737955; doi:10.1371/journal.pone.0187339)

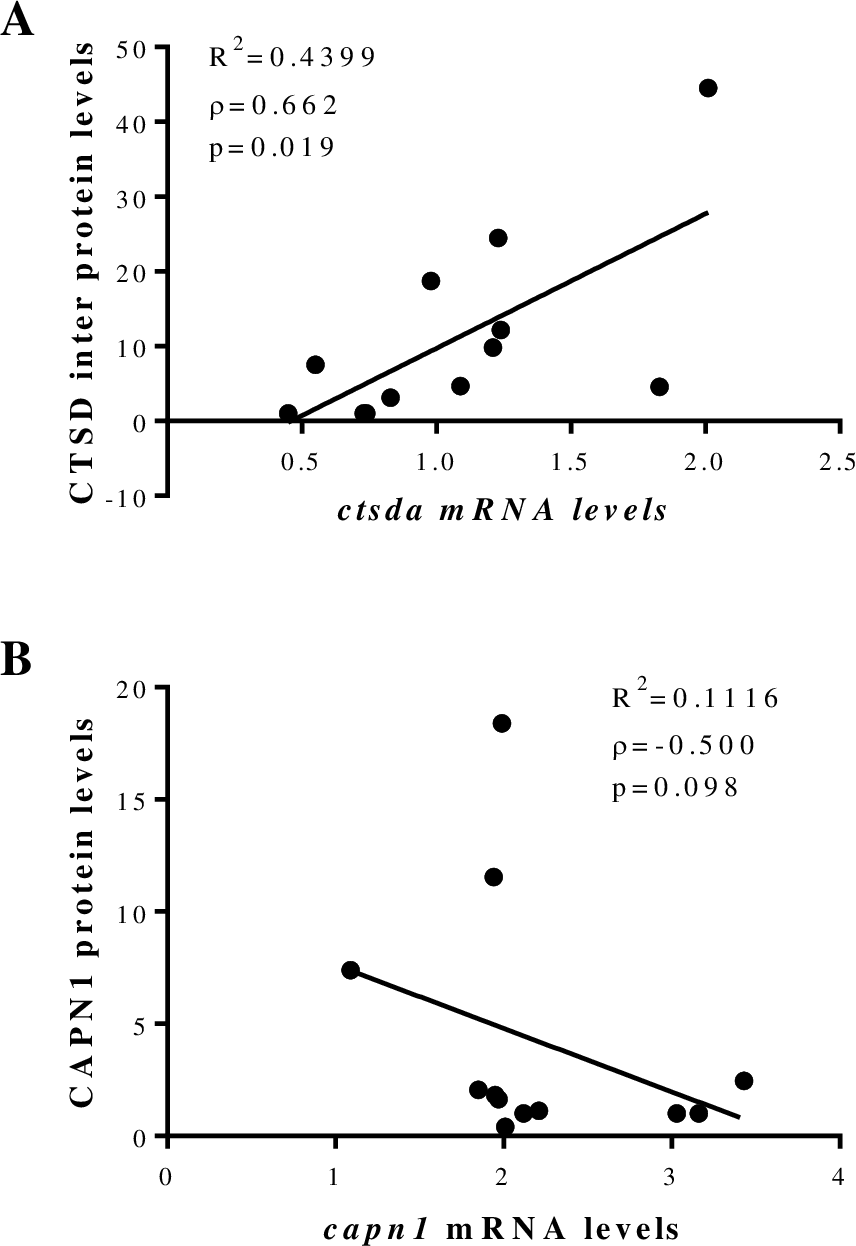

Supplement: S1 Fig — (A) cathepsin Da (ctsda) with cathepsin D intermediate form (CTSD inter), and (B) calpain 1 (capn1) with CAPN1. Data are from n = 3 independent cultures. The R2 of the linear regression, the Spearman’s rank correlation coefficient (ρ) and the p-value are shown. (TIF) [file pone.0187339.s002.tif]
